# Supplementary material for: Transposon mutagenesis screen in Klebsiella pneumoniae identifies genetic determinants required for growth in human urine and serum
Source: eLife. 2024 Aug 27;12:RP88971. doi: 10.7554/eLife.88971 (PMC11349299; doi:10.7554/eLife.88971)
Supplement: Supplementary file 1. — (A) Bacterial strains and plasmids utilized in this study. (B) Primer nucleotide sequences for construction of K. pneumoniae chromosomal mutant strains. (C) Primer nucleotide sequences for enrichment of the transposon junction (TKK_F and TKK_R) and the introduction of an inline barcode for multiplexed sequencing (TKK 6, 7, 8, 9). [file elife-88971-supp1.docx]

**Supplementary File 1a: Bacterial strains and plasmids utilised in this study**

|  | **Description** | **Source** |
| --- | --- | --- |
| Strain |  |  |
| *K. pneumoniae* ECL8 | *K. pneumoniae* isolate derived from NCTC 00418 (Strep^R^) (Amp^R^) (Rif^R^) | (5) |
| *K. pneumoniae* ECL8 *sodA::aph* | *K. pneumoniae* ECL8 with the *sodA* gene replaced with a kanamycin *aph* cassette (Kan^R^) | This study |
| *K. pneumoniae* ECL8 *ytfL::aph* | *K. pneumoniae* ECL8 with the *ytfL* gene replaced with a kanamycin *aph* cassette (Kan^R^) | This study |
| *K. pneumoniae* ECL8 *ompA::aph* | *K. pneumoniae* ECL8 with the *ompA* gene replaced with a kanamycin *aph* cassette (Kan^R^) | This study |
| *K. pneumoniae* ECL8 *fepB::aph* | *K. pneumoniae* ECL8 with the *fepB* gene replaced with a kanamycin *aph* cassette (Kan^R^) | This study |
| *K. pneumoniae* ECL8 *fepD::aph* | *K. pneumoniae* ECL8 with the *fepD* gene replaced with a kanamycin *aph* cassette (Kan^R^) | This study |
| *K. pneumoniae* ECL8 *exbB::aph* | *K. pneumoniae* ECL8 with the *exbB* gene replaced with a kanamycin *aph* cassette (Kan^R^) | This study |
| *K. pneumoniae* ECL8 *exbD::aph* | *K. pneumoniae* ECL8 with the *exbD* gene replaced with a kanamycin *aph* cassette (Kan^R^) | This study |
| *K. pneumoniae* ECL8 *wbbY::aph* | *K. pneumoniae* ECL8 with the *wbbY* gene replaced with a kanamycin *aph* cassette (Kan^R^) | This study |
| plasmid |  |  |
| pKD4 | Used as a template for amplification of the kanamycin aph cassette for the construction of chromosomal mutations. | (6) |
| pACBSCE | Arabinose inducible plasmid that encodes for λ-Red genes: gam, exo and bet llelic exchange vector with arabinose induction | (7) |

**Supplementary File 1b: Primer nucleotide sequences for construction of *K. pneumoniae* chromosomal mutant strains.**

| **Name** | **Primer sequence (5’-3’)** | **Description*** |
| --- | --- | --- |
| ompA_F | GCTAAACTGGGTTACCCGATCACTGACGATCTGGACATCTACACCCG  TCTGGGCGGCATGGTGTAGGCTGGAGCTGCTTC | Forward primer for replacement of *ompA* with the kanamycin *aph* cassette |
| ompA_R | GGGCATAAAAAAAACCCGCCGAAGCGGGTTTTTTTTTATCGGTTATA  ACTTAAGCCGCCGGCTGAGTTACCATATGAATATCCTCCTTAG | Reverse primer for replacement of *ompA* with the kanamycin *aph* cassette |
| ompA_checkF | CAGCTTGGTGCTGGTGCGTTC | Forward check primer for confirmation of the correct disruption of *ompA* |
| ompA_checkR | GCAAAATGTCGAGCCTGAAG | Reverse check primer for confirmation of correct disruption of *ompA* |
| sodA_F | GGACAAACTGCTTACGCGGCGTTAACACTTGAGCCGCTCGACAATAA  TGGAGATGATTATGGTGTAGGCTGGAGCTGTTC | Forward primer for replacement of *sodA* with the kanamycin *aph* cassette |
| sodA_R | GCGAGTCATCAGACTCGCTTCTCTTTTAGCGCAATGCAACCTTATTTTT  TGGCGGCAAAACGCATATGAATATCCTCCTTAG | Reverse primer for replacement of *sodA* with the kanamycin *aph* cassette |
| sodA_checkF | TGCCACAGGATGGCGAACCC | Forward check primer for confirmation of the correct disruption of *sodA* |
| sodA_checkR | GGTCCCCATCGAGACCGAGA | Reverse check primer for confirmation of correct disruption of *sodA* |
| exbB_F | TGTCGTTTTGATATTATTGTGGGCAGATTTTGTGATTATCGTCGTGGAG  ATAGAGCGTGGTGTAGGCTGGAGCTGCTTC | Forward primer for replacement of *exbB* with the kanamycin *aph* cassette |
| exbB_R | CGCCGTTATCGTCCAGGTTTTCATTAAGACGCATCGCCATAGCCGATCA  ACCTACCCGCATATGAATATCCTCCTTAG | Reverse primer for replacement of *exbB* with the kanamycin *aph* cassette |
| exbB_checkF | GCTTTTCTATACCAGCGCACCG | Forward check primer for confirmation of the correct disruption of *exbB* |
| exbB_checkR | GATGGGTTTTTCCGGTCGCGGT | Reverse check primer for confirmation of correct disruption of *exbB* |
| exbD_F | CCAGCGGCGTGAAGCCGGTGCGCAGCGCGCAGAAATTACGGGTAG  GTTGATCGGCTATGGTGTAGGCTGGAGCTGCTTC | Forward primer for replacement of *exbD* with the kanamycin *aph* cassette |
| exbD_R | CAACAAAAAAAGGCCTGCACGCGGCCAGCCTTTGCAGAAACGCAAG  CGGGTTATTTGGCCATATGAATATCCTCCTTAG | Reverse primer for replacement of *exbD* with the kanamycin *aph* cassette |
| exbD_checkF | GTCCTCTGATTCTACGAGGCACG | Forward check primer for confirmation of the correct disruption of *exbD* |
| exbD_checkR | AAATAAACCGGCGCCAGCAGCC | Reverse check primer for confirmation of correct disruption of *exbD* |
| ytfL_F | CACATTTGAGTTATCAACTTCCCTTCCGAGGATCTGGCCTCAACGGT  CAGAAAAGATATGGTGTAGGCTGGAGCTGCTTC | Forward primer for replacement of *ytfL* with the kanamycin *aph* cassette |
| ytfL_R | GGCGGATGGTCATCCGCCCTTAGGAGAGAGAAAAGATTACGCTCAG  GCGTTCTGGCTTTCCATATGAATATCCTCCTTAG | Reverse primer for replacement of *ytfL* with the kanamycin *aph* cassette |
| ytfL_checkF | CTAGCCAGTGTGACAGCCGG | Forward check primer for confirmation of the correct disruption of *ytfL* |
| ytfL_checkR | GCTATCGGCAGAGGGGCGTG | Reverse check primer for confirmation of correct disruption of *ytfL* |
| fepB_F | CACAAAGTTGAAAATGAGACGCATTTATCACCTTTCAAATCAGGAT  GCGATGACGTGGTGTAGGCTGGAGCTGCTTC | Forward primer for replacement of *fepB* with the kanamycin *aph* cassette |
| fepB_R | GCAGGCCGAGTGCCCGTCCTGATGGCGCAGCCCGCGTTAGCCGAAC  AGGCTGGAGAGCATATGAATATCCTCCTTAG | Reverse primer for replacement of *fepB* with the kanamycin *aph* cassette |
| fepB_checkF | TGACGTTTCCATATCATCCTC | Forward check primer for confirmation of the correct disruption of *fepB* |
| fepB_checkR | GCTGGCATTGTAGGCCGGGC | Reverse check primer for confirmation of correct disruption of *fepB* |
| fepD_F | TGAATAAAATCGATAACGATAATTACTATCATTATCATATCAGGGATG  TCAGTTATGGTGTAGGCTGGAGCTGCTTC | Forward primer for replacement of *fepD* with the kanamycin *aph* cassette |
| fepD_R | GCAGACAGCTGGCTATCAGGCGGCGGGACGGGGCAATCACAGGCCA  CCTCCCCGCGGCATATGAATATCCTCCTTAG | Reverse primer for replacement of *fepD* with the kanamycin *aph* cassette |
| fepD_checkF | GCGAGCGATAAAAACGGCGC | Forward check primer for confirmation of the correct disruption of *fepD* |
| fepD_checkR | CCATTAACACCCGCGGCAGC | Reverse check primer for confirmation of correct disruption of *fepD* |
| wbbY_F | ACTACTTCAATTCACTAATATCATAGAAAAGTCTAGGTTACAAAGGA  AGGGTTACAATGGTGTAGGCTGGAGCTGCTTC | Forward primer for replacement of *wbbY* with the kanamycin *aph* cassette |
| wbbY_R | GAAAGTTAATATTGTTTTTGCGGAGCCCTTTCGGGCCCCGAATATTA  CTTTATTTTAACCATATGAATATCCTCCTTAG | Reverse primer for replacement of *wbbY* with the kanamycin *aph* cassette |
| wbbY_checkF | TTACACCATCACCAGCATTAC | Forward check primer for confirmation of the correct disruption of *wbbY* |
| wbbY_checkR | TCCGGCTGAATTCATCCGAAG | Reverse check primer for confirmation of correct disruption of *wbbY* |

*Check primers annealing ~200 bp upstream and downstream of the gene of interest were utilised for confirmation of mutant strains by PCR and sanger sequencing

**Supplementary File 1c:** **Primer nucleotide sequences for enrichment of the transposon junction (TKK_F and TKK_R) and the introduction of an inline barcode for multiplexed sequencing (TKK 6, 7, 8, 9)**

| **Name** | **Primer sequence (5’-3’) *** |
| --- | --- |
| TKK 6.1 | AATGATACGGCGACCACCGAGATCTACACTCTTTCCCTACACGACGCTCTTCCGATCTCGTACGAGCTTCAGGGTTGAGATGTGTA |
| TKK 6.3 | AATGATACGGCGACCACCGAGATCTACACTCTTTCCCTACACGACGCTCTTCCGATCTTACGTAAGCTTCAGGGTTGAGATGTGTA |
| TKK 7.2 | AATGATACGGCGACCACCGAGATCTACACTCTTTCCCTACACGACGCTCTTCCGATCTGCTAGCTAGCTTCAGGGTTGAGATGTGTA |
| TKK 7.4 | AATGATACGGCGACCACCGAGATCTACACTCTTTCCCTACACGACGCTCTTCCGATCTTAGCTAGAGCTTCAGGGTTGAGATGTGTA |
| TKK 8.2 | AATGATACGGCGACCACCGAGATCTACACTCTTTCCCTACACGACGCTCTTCCGATCTATGCATGCAGCTTCAGGGTTGAGATGTGTA |
| TKK 8.3 | AATGATACGGCGACCACCGAGATCTACACTCTTTCCCTACACGACGCTCTTCCGATCTCATGCATGAGCTTCAGGGTTGAGATGTGTA |
| TKK 8.4 | AATGATACGGCGACCACCGAGATCTACACTCTTTCCCTACACGACGCTCTTCCGATCTCGTACGAGCTTCAGGGTTGAGATGTGTA |
| TKK 9.2 | AATGATACGGCGACCACCGAGATCTACACTCTTTCCCTACACGACGCTCTTCCGATCTATCGATCGAAGCTTCAGGGTTGAGATGTGTA |
| TKK 9.3 | AATGATACGGCGACCACCGAGATCTACACTCTTTCCCTACACGACGCTCTTCCGATCTTCGATCGATAGCTTCAGGGTTGAGATGTGTA |
| TKK 9.4 | AATGATACGGCGACCACCGAGATCTACACTCTTTCCCTACACGACGCTCTTCCGATCTCGATCGATCAGCTTCAGGGTTGAGATGTGTA |
| TKK_F | ACCTGCAGGCATGCAAGCTTCAGG |
| TKK_R | GACTGGAGTTCAGACGTGTGCTCTTCCGATC |

*The expected inline barcode is underlined
